# Get to grips with motivation: Slipping and gripping movements are biased by approach-avoidance context

**DOI:** 10.3389/fpsyg.2022.989495

**Published:** 2022-10-18

**Authors:** Sofie Johanna Nilsson, David Meder, Kristoffer Hougaard Madsen, Ivan Toni, Hartwig Roman Siebner

**Affiliations:** ^1^Danish Research Centre for Magnetic Resonance, Centre for Functional and Diagnostic Imaging and Research, Copenhagen University Hospital – Amager and Hvidovre, Copenhagen, Denmark; ^2^DTU Compute, Technical University of Denmark, Lyngby, Denmark; ^3^Centre for Cognitive Neuroimaging, Donders Institute for Brain, Cognition, and Behavior, Radboud University Nijmegen, Nijmegen, Netherlands; ^4^Department of Neurology, Copenhagen University Hospital Bispebjerg, Copenhagen, Denmark; ^5^Faculty of Medical and Health Sciences, Institute for Clinical Medicine, University of Copenhagen, Copenhagen, Denmark

**Keywords:** approach avoidance behavior, approach avoidance task (AAT), appetitive and aversive effects, healthy volunteers, grip force control

## Abstract

People are better at approaching appetitive cues signaling reward and avoiding aversive cues signaling punishment than vice versa. This action bias has previously been shown in approach-avoidance tasks involving arm movements in response to appetitive or aversive cues. It is not known whether appetitive or aversive stimuli also bias more distal dexterous actions, such as gripping and slipping, in a similar manner. To test this hypothesis, we designed a novel task involving grip force control (gripping and slipping) to probe gripping-related approach and avoidance behavior. 32 male volunteers, aged 18–40 years, were instructed to either grip (*“approach”*) or slip (*”avoid”*) a grip-force device with their right thumb and index finger at the sight of positive or negative images. In one version of this pincer grip task, participants were responding to graspable objects and in another version of the task they were responding to happy or angry faces. Bayesian repeated measures Analysis of variance revealed extreme evidence for an interaction between response type and cue valence (Bayes factor = 296). Participants were faster to respond in affect-congruent conditions (*“approach appetitive,” “avoid aversive”*) than in affect-incongruent conditions (*“approach aversive,” “avoid appetitive”*). This bias toward faster response times for affect-congruent conditions was present regardless of whether it was a graspable object or a face signaling valence. Since our results mirror the approach and avoidance effects previously observed for arm movements, we conclude that a tendency favoring affectively congruent cue-response mappings is an inherent feature of motor control and thus also includes precision grip.

## Introduction

Motivation is an important driving force of motor control (Reeve, 2014). People generally prefer to approach appetitive stimuli and to avoid aversive stimuli (Solarz, 1960; Dickinson and Balleine, 2002; Roelofs et al., 2009; Krieglmeyer et al., 2010; Volman et al., 2011). A range of Approach Avoidance Tasks (AATs) have been used to study the impact of appetitive and aversive cues on approach and avoidance behavior (Solarz, 1960; Chen and Bargh, 1999; Heuer et al., 2007). In a joystick version of the AAT, participants are instructed to move a joystick at the sight of appetitive or aversive stimuli (Heuer et al., 2007; Roelofs et al., 2009). In congruent trials, they are instructed to pull the joystick toward themselves at the sight of appetitive stimuli, and to push the joystick away from themselves as a response to aversive stimuli. In incongruent trials, instructions are reversed. While participants can produce the required response in both congruent and incongruent trials, they show longer reaction times for incongruent trials compared to congruent trials (Roelofs et al., 2009; Tyborowska et al., 2016). The delay in response time for incongruent trials has been attributed to inherent motivational biases. Positive and negative stimulus evaluations elicit automatic Pavlovian response tendencies that compete with the instructed response in incongruent trials (Chen and Bargh, 1999; Roelofs et al., 2009). Analogous interference effects to the above mentioned ones have indeed been demonstrated when agents were asked to physically walk toward or away from a happy or angry faces (Stins et al., 2011).

If approach and avoidance are hardwired response tendencies, they should generalize across movements and not be limited to whole-limb or whole-body movements. Most studies have used ecologically valid approach and avoidance responses, such as arm flexion vs. extension movements that would also move objects closer or further in the real world (Roelofs et al., 2009; Volman et al., 2011). Other AATs only required a simple button press vs. refraining from pressing a button (Guitart-Masip et al., 2014) or producing button presses to move a manikin on a computer screen toward or away from aversive or appetitive stimuli (De Houwer et al., 2001). However, it is unclear whether an interference effect also exists for manipulative movements such as manual tasks requiring a pincer grip. Indeed, control of grip force is relevant for approach-avoidance control within peripersonal space, when gripping or slipping an object.

This study was designed to test the hypothesis that appetitive or aversive stimuli also bias more distal dexterous actions, such as gripping and letting go of stimuli (henceforth referred to as “slipping”), in a similar manner as it had been previously shown for whole-limb or whole-body movements. To this end, we designed a novel pincer grip task which involved fine force control with the tip of the thumb and index finger (gripping and slipping) to capture approach and avoidance biases to aversive and appetitive stimuli. We predicted that such inherent bias would result in an incongruency effect with longer response initiation times for incongruent cue-response conditions (approach aversive cues and avoid appetitive cues) compared to congruent cue-response conditions (approach appetitive and avoid aversive cues).

These basic motivational tendencies to approach appetitive and to avoid aversive objects, persons or activities are thought to be coupled to the valence of stimuli *via* hard-wired, Pavlovian biases (Eder et al., 2013). If so, tightly coupled stimulus-response relationships that are commonly experienced in the environment may be needed to establish these approach-avoidance biases, and they might not generalize to valence-action mappings that are not directly associated in real life. Outside the realm of approach-avoidance behavior, previous studies have shown an impact of the motivational context on grip force control. Healthy participants exerted stronger grip forces depending on the prospect of reward (Pessiglione et al., 2007; Klein-Flügge et al., 2016). In addition, the valence-action incongruency effect has been consistently evoked by different categories of action cues, such as pictures of faces, emotional words (Solarz, 1960), and voices (Koch et al., 2020). Hence, one may expect that a tight ecological relatedness between the valent action cue and the triggered action might not be needed to trigger an approach-avoidance bias. To address this question, our novel pincer grip task featured two types of visual action cues. One category was directly related to a gripping-slipping context (e.g., pictures of graspable objects), while the other category was only indirectly related to a gripping-slipping (e.g., pictures of emotional faces). We expected to find that both cue categories would give rise to an approach-avoidance bias rather than a selective approach-avoidance bias for graspable objects. A third aim of this study was to explore whether the postulated approach-avoidance bias was modulated by individual, personality-related, factors.

## Materials and methods

### Participants

Thirty two healthy male participants age 27.1 ± 6.2 (mean ± onefold standard deviation) were recruited *via* the recruiting website “forsoegsperson.dk” to participate in the study. We included only male participants to avoid potential cyclic influences on approach avoidance behavior that were not controlled for Feltenstein and See (2007). All participants had normal or corrected-to-normal vision. Written informed consent was obtained prior to participation. One participant was excluded due to technical problems resulting in poor data quality. The data of the remaining 31 participants were included in the final analysis. The study was approved by the local ethics committee (H-3-2014-109) and the national data protection agency (VD-2014-332).

### Experimental procedures

Each participant took part in a single experimental session in which they performed two versions of the AAT. We tested two versions of the AAT, because we wanted to explore whether the stipulated incongruency effect generalizes not only across effectors but also across visual object categories, as it has previously been shown in a study using the joystick AAT together with emotional voices (Koch et al., 2020). The two versions of the AAT were identical in design apart from the type of stimuli (objects or faces) that cued the response. In addition, the AAT version with objects included additional task blocks in which neutral stimuli were presented.

**FIGURE 1:**
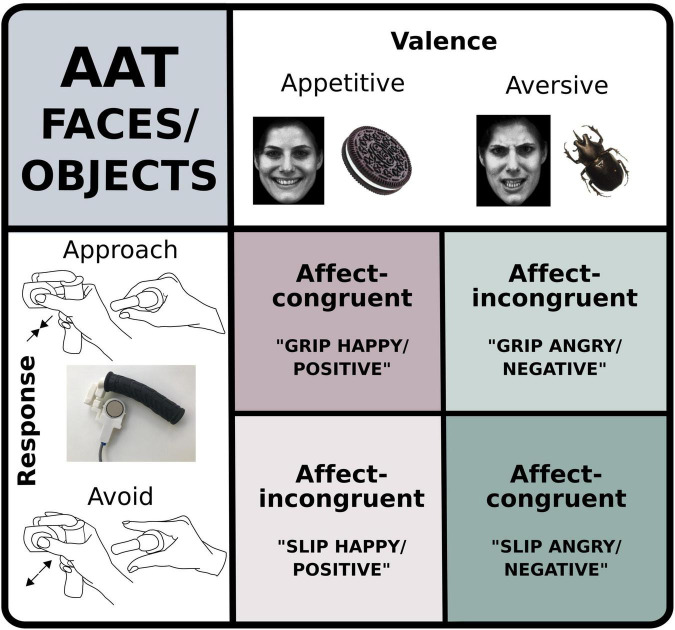
Graphic representation of the two versions of the modified approach avoidance task (AAT). In one version, participants viewed happy and angry faces and in the other one they were viewing appetitive or aversive objects. At the sight of the images, they were instructed either to grip or slip a custom-made force device (see inset bottom left). In affect-congruent conditions participants were instructed to grip the device at the sight of happy faces or positive images and to slip the device at the sight of angry faces or negative images. In affect-incongruent conditions instructions were reversed.

Our experimental paradigm probed gripping-slipping behavior cued by graspable objects or faces (Figure 1). We created a novel set of graspable stimuli as cues, in addition to face images, that have previously shown to evoke approach and avoidance conflict as represented by arm movements (Tyborowska et al., 2016).

**FIGURE 2:**
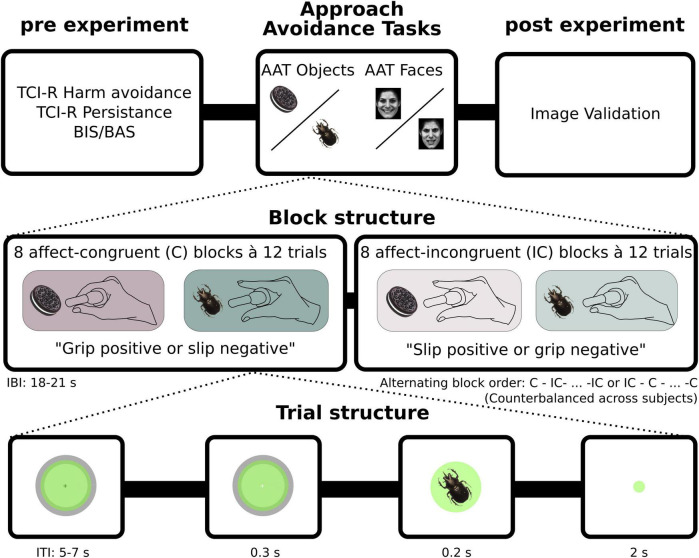
Experimental structure. Prior to performing the two version of the approach avoidance task (AAT) (with Faces or Objects as stimuli) participants filled out questionnaires and received training on the task. The AAT-Faces consisted of 16 blocks, whereof 8 were with affect-congruent instructions (grip positive and slip negative) and 8 were with affect-incongruent instructions (slip positive and grip negative). The AAT-Objects task had an additional 4 blocks with neutral cues (not depicted here, see methods). Each block in turn consisted of 12 trials, with equally many positive as negative images presented in a pseudorandomized order. Each trial began with participants exerting force at baseline level (2% of MVC). Once baseline level was obtained and participants had held it constant for minimum 1 s, the circle on the screen, giving visual feedback of force input, changed color from green to blue, indicating the start of the trial. Shortly thereafter, stimuli appeared on the screen for 0.2 s. Participants were instructed to respond as fast and as accurately as possible according to block instructions.

The timeline of the experimental procedures is illustrated in Figure 2. In the beginning of each session, written informed consent was obtained and participants then filled out three questionnaires: First, the BIS/BAS scale (Carver and White, 1994) which is designed to measure two motivational systems underlying behavior. The behavioral inhibition system (BIS) is thought to drive the motivation to avoid aversive outcomes, while the behavioral activation system motivates the approach of something desired. The BIS is assessed with one scale only, while the BAS comprises three subscales: BAS Drive (goal-pursuit), BAS Fun seeking (find and approaching novel rewards), and BAS Reward responsiveness (sensitivity to pleasant reinforcers). The other two questionnaires were two subscales of the Temperament and Character Inventory TCI-R (Cloninger, 1994): TCI-R Harm avoidance, which captures a personality trait with facets such as excessive worrying, pessimism, shyness and being fearful. TCI-R Persistence is characterized by persistent behavior in spite of fatigue or frustration.

Participants were thereafter seated in front of a computer approximately 71 centimeters away from the screen for training and performing the AAT. To familiarize participants with the task, two blocks of each version of the task were administered in a pseudorandomized order prior the experiment. The stimuli shown during practice were different to the ones used for subsequent testing. Each session ended with an image validation task, in which participants gave valence and arousal ratings on all stimuli in the AAT version with objects. For each image, they were asked the following two questions: “What was your initial reaction to this image?” (Valence; 0 very negative, 50, neutral, 100 very positive, and “How much did this image affect you?”) (Arousal; 0: not at all, 100 very much).

### Experimental task

Each participant performed both versions of the modified AAT (Figures 1, 2) with the order counterbalanced between participants. In one of the task versions (“AAT-Objects”) stimuli consisted of images of various objects, such as berries or insects and in the other version (“AAT-Faces”) participants were viewing images of angry or happy faces. During the AAT, participants were instructed to respond to images according to their emotional valence (“positive” or “negative” for AAT-Objects, and “happy” or “angry” for AAT- Faces). Motor responses were recorded using a custom-made grip force device allowing participants to either grip or slip a flat plastic block with two pressure sensors on either side at the top of the device, which was held with a pinch grip between thumb and index finger. The device could be held around the handle with the remaining fingers even when entirely slipping the top part (see Figure 1).

Participants held the device in their dominant hand and were instructed to either grip or slip at the sight of different images. In affect-congruent conditions, participants were instructed to grip the device (“approach”) when they saw a happy face or something positive) and to slip completely (“avoid”) at the sight of an angry face or something negative. In affect-incongruent conditions, instructions participants were instead instructed to press (“approach”) at the sight of an angry face or something negative and to let it go (“avoid”) when a happy face or a positive image was shown (Figure 1). In the initial phase of each trial, participants were instructed to exert a low baseline force [2% of their Maximal Voluntary Contraction (MVC) measured before the start of the experiment]. Visual feedback about the applied pressure was provided in the form of a green circle, increasing in size relative to the force exerted (Figure 2). A gray ring indicated how large the green circle had to be in order to apply baseline force (2% MVC). The gray ring’s outer and inner rim was at baseline force ± 25% of baseline force. Participants were instructed to exert the amount of force required to keep the green circle between inner and outer margin of the gray ring. Once the participant had filled out the circle and kept their force level constant for 1°s, the circle fill color changed from green to blue, indicating the start of a trial. A fixation cross appeared briefly for 100 ms, then only the blue circle was shown for another 300 ms followed by the presentation of the face or object cue for 200 ms.

Participants had 2 s to respond to the cue. The size of the circle constantly provided online feedback about the exerted force. They were instructed to respond as fast and as accurately as possible. A response was considered valid if the force input deviated more than ± 30% from baseline force (2% of MVC). If no response was given, participants were prompted by a message on the screen to do so and then had additionally 2 s to respond. After a response was given (or after the 4 s had passed), the circle turned green again, indicating that participants had to return to baseline force levels again. After 2–3 s (random uniform distribution), a new trial started.

The task had a blocked design with congruent and incongruent blocks following each other in an alternating order. The type of starting block (congruent or incongruent) was alternated among participants in a counterbalanced order. The AAT version using faces consisted of 8 congruent and 8 incongruent blocks with 12 trials per block. The AAT version using graspable objects consisted of 8 congruent and 8 incongruent blocks with 12 trials per block, and additional 4 neutral blocks with 12 trials each. In these four additional blocks, participants were instructed to categorize neutral stimuli (such as a button or a piece of stone) according to color. A neutral block always followed two congruent and two incongruent blocks. These neutral blocks were included to obtain baseline measurements of grip and slip in a neutral context, but not incorporated in the final analysis.

### Experimental stimuli

The stimuli used in the AAT-Faces were collected from different databases (Ekman, 1976; Matsumoto and Ekman, 1988; Lundqvist et al., 1998; Martinez and Benavente, 1998). The stimuli set consisted of 36 models (18 male) each with a happy and an angry facial expression. The faces were trimmed from hair and non-facial contours, matched for color and brightness, and presented in gray scale against a black background. The stimuli set used in the AAT-Objects was collected and produced on site. The stimuli set consisted of 40 graspable objects (16 appetitive, 16 aversive, and 8 neutral). All stimuli were presented on a computer screen at a visual angle of 6 × 8 degrees. The experiment was programed and presented on a PC using Psychopy (version 1.74.00).

### Data processing

**FIGURE 3:**
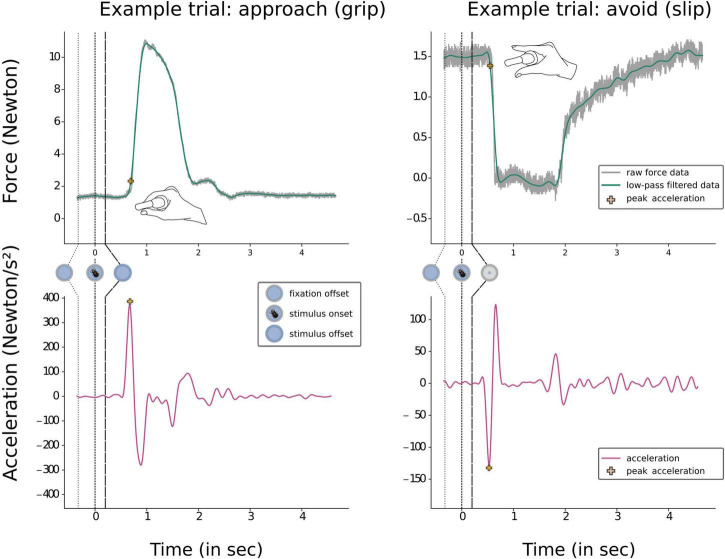
Examples of time force curves during a grip and a slip trial. The two upper boxes depict raw force data and the subsequent low pass filtered data, with the point of peak acceleration marked by a yellow cross. The time point of peak acceleration was used to extract our first dependent variable of interest; response initiation time (RIT). Prior to responding participants were pressing the device at a baseline level (2% of MVC). The lower two boxes illustrate the second derivate of the time force curve, force acceleration. Again, the peak acceleration is marked by a yellow cross. Here, the magnitude of peak acceleration was used to extract or second dependent variable of interest: peak response acceleration (PRA).

Force data were recorded with a PicoLog 1216 data logger (Pico Technology) throughout the whole experiment at a sample rate of 500°hz and stored for subsequent analysis. All force data analysis was done using Python (Version 2.7). Data from the two different input channels (thumb and index finger) was averaged and the resulting force curve was smoothed using a linear phase Type II finite impulse response (FIR) low-pass filter (200 taps, cut-off frequency 5 Hz). The response window for a given trial was defined as 4 s from 100 ms after stimulus onset. Within the response window, the response initiation time (RIT) was defined as the time of the first peak of the second derivative (acceleration) of the smoothed force curve. We also extracted the magnitude of this first peak (peak response acceleration, PRA) (Figure 3). Responses used in subsequent analyses were defined in two steps relative to the individual baseline force. During the experiment, the on-line force level had to deviate from baseline more than 30% to be considered as “response.” If no such response was detected, participants were prompted to respond and were given additionally 2°s to do so. For off-line analysis, the definition of the type of response (grip or slip) was based on the smoothed force curve. Here, force input during the first 100 ms after stimulus onset (i.e., where no valid response can occur yet) was used as a baseline measure against which subsequent force input during the response window was compared to detect and classify responses. The first deviation exceeding ± 30% of baseline force compared to the mean force during the first 100 ms of stimulus presentation was used for classification: An increase in force of ≥ 30% of baseline level was classified as a grip response. If force input decreased more than 30% relative to baseline, it was classified as a slip response.

In addition to grip and slip definition, responses recorded after a response reminder was coded as a “late response.” In trials where participants responded with more than one type of response, for example if they changed their mind or tried to self-correct, the first response was used for response classification.

### Statistical analysis

R Studio (Version 1.0.136) and Matlab (R2020a) were used for subsequent dataset preparation (removal of outliers and incorrect responses). All descriptive and inferential statistics were made using JASP (Version 0.11.1). We wished to investigate how fast participants initiated a response to differently valenced stimuli, depending on the congruency of required responses. Our behavioral analysis therefore focused on response initiation time (RIT) which was defined as primary variable of interest. For each participant, RIT outliers were defined by a 1.5 interquartile range (IQR). Observations more than 1.5 IQR below quartile 1 and more than 1.5 IQR above quartile 3 were thus not considered for further analysis. Remaining trials with RIT below 150°milliseconds or above 1.2 s as well as incorrect responses were excluded from further analysis. In the AAT task using graspable objects, three object stimuli were on average rated opposite to our valence categorization and hence had to be removed from further analysis (see further details below). In total, 23.2% of all trials were excluded. Using the remaining trials, mean RIT (in milliseconds) was calculated in each participant for each combination of the three experimental factors (Task and Response and Affect).

To examine the effect on RIT of each of the factors Task (Faces, Objects), Response (grip, slip), and Valence (Appetitive, Aversive) we carried out a Bayesian repeated measures ANOVA with the average RIT for each participant within each of the eight condition combinations.

Prompted by a suggestion made by one of the reviewers, we conducted a follow-up analysis to investigate if the gender of faces influenced RIT. To this end, we carried out a Bayesian repeated measures ANOVA testing the effect on RIT on each the three factors Gender (Female, Male), Response (Grip, Slip), and Valence (Appetitive, Aversive).

Our choice of Bayesian statistics was motivated by the advantages it has compared to frequentist statistics [e.g., the ability to provide support for both H1 and H0, avoiding an arbitrary decision threshold (*p*-value) toward a more gradual description of evidence (Bayes factors), “inbuilt” penalization of more complex models that include more factors etc., (Keysers et al., 2020; Schad et al., 2022)]. Bayes factors are classified according to the scheme of Jeffreys (Jeffreys, 1998; Keysers et al., 2020) [Bayes Factor (BF) = 1–3: anecdotal evidence, BF = 3–10: moderate evidence, BF = 10–30: strong evidence, BF = 30–100: very strong evidence, BF > 100: extreme evidence]. BF_10_ denotes evidence in favor of a given model against the null model, BF_01_ denotes evidence in favor of the null model (Keysers et al., 2020; Schmalz et al., 2021).

If the interference effect were dependent on the affordance of the stimulus presented, we would expect trials depicting graspable objects to be more sensitive to the gripping and slipping response bias compared to trials depicting faces. This would result in a three-way interaction between type of task stimuli, response and valence. In addition to investigating support for the winning model, we employed model averaging to investigate analysis of effect for each of the factors alone. In model averaging, the evidence in favor of including a factor into the model is calculated by combining evidence across all models that include that factor. Hence, all models are considered in a set, by summing the posterior probability for all models that include the effect, and then comparing the posterior inclusion odds to the prior inclusion odds. The resulting inclusion BF (BF *_incl_*) thus denotes the support for including a specific factor in the model averaged across all the considered models (van den Bergh et al., 2020).”

In a follow-up analysis, we went on to investigate individual responses in order to see if there was a consistent pattern of approach and avoidance tendencies across participants. To this end, we first calculated RIT differences, henceforth referred to as “deltas,” between the different valence conditions within the same response type, and thereafter plotted slopes between the two resulting deltas: For each participant, we calculated RIT for each of the four conditions (*approach appetitive*, *approach aversive*, *avoid appetitive*, and *avoid aversive*) for each of the two task versions, respectively (“AAT-Faces” and “AAT-Objects”), as well as for the two task versions combined. For both responses (*approach* and *avoid*), we then subtracted the mean RIT for the negative condition from the positive condition. If our hypotheses regarding incongruent images giving rise to larger RIT were correct, participants would show negative deltas for “*approach appetitive–approach aversive*” and positive deltas for “*avoid appetitive–avoid aversive*.” We calculated mean valence rating as well as mean RIT for each stimulus and performed a Bayesian correlations pair analysis on the two variables to investigate the relationship between valence ratings and RIT.

Furthermore, we also used error rates as outcome measure since they have been shown to be affected by congruency (Volman et al., 2011; Bramson et al., 2020a) and we expected them to be similarly affected by our experimental manipulations. The same repeated measures ANOVAs, including covariates, that were carried out with RIT as dependent variable, were run with error rates as dependent variable.

To investigate the effect of personality factors obtained from our three questionnaires (BIS/BAS, TCI-R Harm Avoidance, and TCI-R Persistence), we included them as covariates in our Bayesian repeated measures ANOVA model. All three BAS subscales (Drive, Fun seeking, Reward sensitivity) were modeled separately. As illustrated in Figure 3, grip-force measures provide a rich dataset, with the possibility to examine several aspects of a response. To explore whether other dependent variables reflect different aspects of approach-avoidance behavior not revealed by RIT or error rates, we included additional exploratory analyses with additional dependent variables (the amplitude of the acceleration [peak response acceleration (PRA)] as a proxy for “response motivation” (Mazzoni et al., 2007), the variability in RIT as well as PRA, and finally, upon reviewer suggestion, the area under the curve, see Supplementary material).

## Results

### Response initiation time

**FIGURE 4:**
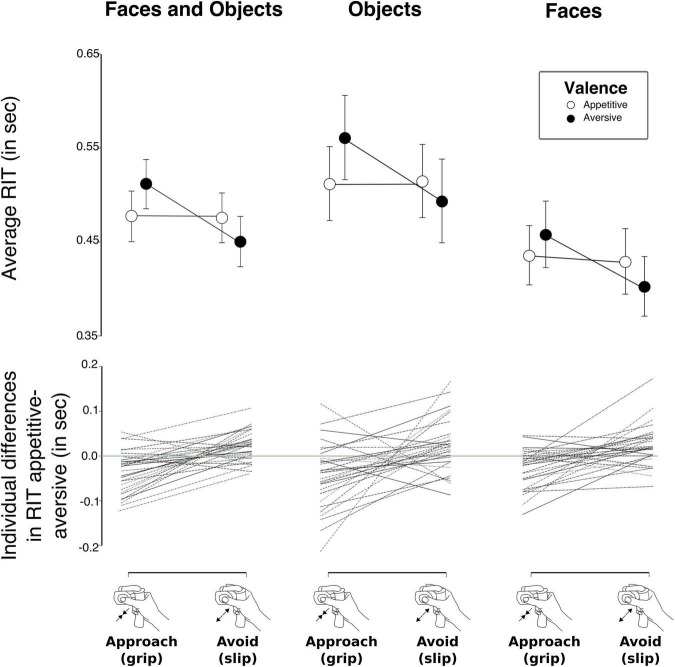
**(Upper row)** Mean Response Initiation Times (RIT) for the two versions of our approach avoidance task (AAT) combined (left) as well as for the two versions of the AAT separately (middle and right). Across versions, participants were faster to initiate an “approach” response to appetitive stimuli and to initiate an “avoid” response to aversive stimuli than vice versa. Error bars denote 95% Bayesian credible interval. **(Lower row)** Individual deltas (RIT differences) for response initiation times in the approach appetitive–approach aversive conditions as well as for response initiation times in the avoid appetitive–avoid aversive conditions. Dotted lines connect the two deltas for each individual.

Mean RIT for the four conditions *(approach appetitive, approach aversive, avoid aversive*, and *avoid appetitive*) are shown for the two versions of the task (“AAT-Faces” and “AAT-Objects”) in Table 1. Results from our Bayesian repeated measures ANOVA showed that the model with the strongest support for explaining our data was the one including the main effects of Task (objects vs. faces), Response (grip vs. slip), and Valence (appetitive vs. aversive) and the interaction between Response and Valence (Table 2). With a Bayes factor of BF_10_ = 1.752e + 28, there was extreme support for this model compared to a null-model with only a subject factor (see methods section for Bayes Factor evidence categories). The interaction between response and valence was due to longer RITs for incongruent trials compared to congruent trials (Figure 4). Accordingly, model averaging revealed extreme evidence for the presence of an effect of the factors Task (inclusion Bayes factor BF_*incl*_ = ∞), Response (BF_*incl*_ = 53396.70), and the Valence x Response interaction (BF_*incl*_ = 296.42) and very strong evidence for the factor Valence (BF_*incl*_ = 55.58). All other factors had BF_*incl*_ < 1. As mentioned above, by model averaging, the evidence for including a factor into the model is calculated by combining across all models including that factor. The extreme evidence for the presence of an interaction effect between Valence and Response thus confirmed our hypothesis that approach and avoidance biases are reflected in gripping responses. Concerning a three-way interaction between Valence, Response, and Task, the evidence is in favor of the null hypothesis that there is no such interaction (BF_01_ = 4.467, moderate evidence). In other words, the statistical evidence did not indicate that approach and avoidance biases would be more pronounced for graspable objects compared to non-graspable faces. Results from investigating individual mean RIT differences between appetitive and aversive stimuli are illustrated in Figure 4. A majority of participants had negative deltas for RIT_*approach appetitive*_ – RIT_*approach aversive*_ and positive deltas for RIT_*avoid appetitive*_ – RIT_*avoid aversive*_. In addition, a majority of participants had positive slopes between the two deltas.

**TABLE 1:** Mean response initiation times (RIT), peak response acceleration (PRA), and error rates for each of the four conditions for the two versions of the task.

	AAT-faces				AAT-objects			
	Approach appetitive	Approach aversive	Avoid appetitive	Avoid aversive	Approach appetitive	Approach aversive	Avoid appetitive	Avoid aversive
Mean RIT (sec)	0.437	0.46	0.43	0.403	0.517	0.566	0.52	0.499
SD	0.088	0.098	0.097	0.088	0.107	0.122	0.106	0.121
Mean PRA (Newton^2^)	51.934	54.404	–8.927	–8.307	44.21	47.309	–7.284	–8.063
SD	40.553	40.664	7.357	4.314	37.234	35.478	2.904	4.005
Error rates (%)	19.5	20.8	12.5	8.2	21.8	32	16	16.2
SD	15.7	25.8	7.4	7.6	17.4	13.8	11.2	11.2

**TABLE 2:** Model winners and BF_incl_ for the different factors included in the winning model for each of the outcome variables: response initiation time (RIT), RIT (for AAT faces only), peak response acceleration (PRA), variance of RIT (Var_RIT), variance of PRA (Var_PRA), error rates, and area under the curve (AUC).

Dependent variable	Best model	BF_10_ = best model	Factor 1	Factor 2	Factor 3	Factor 4	Factor 5	Factor 6	Factor 7	Factor 8	Factor 9	Factor 10
RIT	Task + response + valence + response*valence	1.752e +28	Task	Response	Valence	Task* response						
			BF_incl_ (infinity)	BF_incl_ = 53396.699	BF incl 55.582	BF_incl_ = 55.582						
RIT (faces only)	Response + valence + gender +	6.288e +6	Response	Valence	Gender	TCI-R harm avoidance	TCI-R persistence	BAS drive	BAS fun seeking	Response* valence	Response* gender	Valence* gender
	TCI-R harm avoidance + TCI-R persistence		BF_incl_ = 9827.799	BF_incl_ = 9897.288	BF_incl_ = 349.297	BF_incl_ < 1	BF_incl_ < 1	BF_incl_ < 1	BF_incl_ < 1	BF_incl_ = 316.506	BF_incl_ = 4.700	BF_incl_ = 1121.952
	BAS drive + BAS fun seeking + response*valence +											
	Response*gender + valence*Gender											
PRA	Response + BAS fun seeking	>100	Response	BAS fun seeking								
			BF_incl_ (infinity)	BF_incl_ = 17.029								
Var_RIT	Task	3.861e +9	Task									
			BF_incl_ = 2.471e +9									
Var_PRA	Task + response + BAS fun seeking	503,319	Task	Response	BAS fun seeking							
			BF_incl_ = 2.415	BF_incl_ = 19.287	BF incl 2.838							
Error rates	Task + response + valence + TCI-R persistence +	1.004e +11	Task	Response	Valence	TCI-R persistence	Task* valence	Response* valence				
	Task*valence + response*valence		BF_incl_ = 654.579	BF_incl_ = 4.537e +8	BF incl 1.996	BF_incl_ = 2.838	BF_incl_ = 2.993	BF_incl_ = 5.535				
AUC	Response + BAS drive + BAS fun seeking +	3.963e +6	Response	BAS drive	BAS fun seeking	BAS reward responsiveness						
	BAS reward responsiveness		BF_incl_ = 1.511e+6	BF_incl_ < 1	BF_incl_ = 1.556	BF_incl_ = 0.857						

Results from the follow-up analysis testing whether the gender of the face stimulus had an effect on approach-avoidance behavior showed that the model with the strongest support for explaining our data was the one including the main effects of Response (grip vs. slip), Valence (appetitive vs. aversive), Gender (female vs. male), TCI-R Harm avoidance, TCI-R Persistence, BAS Drive, BAS Fun seeking and the interactions between Response and Valence, Response and Gender as well as between Valence and Gender (Supplementary Figure 1). With a Bayes factor of BF_10_ = 6.288e + 6, there was extreme support for this model compared to a null-model with only a subject factor. Accordingly, model averaging revealed extreme evidence for the presence of an effect of the factors Response (inclusion Bayes factor BF_*incl*_ = 9827.799), Valence (BF_*incl*_ = 9897.288), Gender (BF_*incl*_ = 349.297), Response x Valence (BF_*incl*_ = 316.506) and Valence x Gender (BF_*incl*_ = 1121.952) and anecdotal support for Response x Gender (BF_*incl*_ = 4.700) and Response x Valence x Gender (BF_*incl*_ = 3.309), and BIS (BF_*incl*_ = 1.382). All other factors had BF_*incl*_ < 1 (Table 2). Thus, even though four covariates were part of the winning model and thus contributed to explaining RITs in the different conditions, there were apparently many well-performing models that did not include these covariates, thus providing little support for including these covariates in the average model, resulting in BF_*incl*_ < 1. The three interactions were driven by generally fast avoid responses to female faces and generally slow approach responses to male faces (irrespective of valence). Approaches to female faces were slower for angry compared to happy faces, while avoid responses to male faces were slower for happy compared to angry faces.

None of the additional dependent variables, including RIT variability, peak response acceleration PRA and PRA variability, and AUC, used in our exploratory analyses showed evidence for an interaction effect between Response and Valence (see Supplementary material).

### Error rates

Another Bayesian repeated measures ANOVA was carried out using error rates as dependent variable and with scores on all questionnaires as covariates. Mean error rates for the four conditions (*approach appetitive, approach aversive, avoid aversive*, and *avoid appetitive*) are shown for the two versions of the task versions (“AAT-Faces” and “AAT-Objects”) in Table 1. The model with the strongest support for explaining our data (BF_10_ = 1.004e + 11) was the one including the factors Task, Response, Valence, the covariate TCI-R Persistence as well as the interaction terms Task*Valence and Response*Valence (see Table 2). Model averaging revealed extreme evidence for an effect of the factors Task and Response (BF_incl_ = 654.579 and 4.573e + 8, respectively), whereas it only was anecdotal for Valence (BF_incl_ = 1.996), moderate for the two interaction terms (BF_incl_ = 2.993 and 5.535, respectively), and not supported for TCI-R Persistence (BF_incl_ = 0.726). The interaction between response and valence was due to more errors committed in incongruent trials [mean 16.4% (7.1)] compared to congruent trials [mean 20.3% (8.8)]. The main effect of Task was due to more errors committed in AAT-Objects [mean 21.5% (± 9.7)] compared to AAT-Faces trials [mean 15.2% (± 8.4)]. The interaction effect between Task and Valence was reflected in the fact that in AAT-Faces, participants made more errors in response to appetitive images [mean 15.9% (± 9.2)] compared to aversive images [mean 14.5% (± 8.3)] whereas in AAT-Objects they made more errors in response to aversive stimuli [mean 24.1% (± 9.6)] compared to appetitive images [mean 18.9% (± 12.1)]. Follow-up exploratory analysis revealed a negative correlation between scores on TCI-R and error rates for all conditions across both tasks. The only correlation that gained support (anecdotal, BF_10_ 1–3) was the one between error rates in AAT Faces for the condition *avoid appetitive* (see Supplementary Table 1).

### Valence and arousal stimuli ratings

Mean valence and arousal ratings (0–100) were calculated for each category of object stimuli; aversive, neutral, and appetitive. On average, aversely categorized images were perceived as negative [mean rating 21.3 (20.8)] and appetitively categorized images were perceived as positive [mean rating 77.2 (23.5)]. Neutrally categorized images were rated as slightly positive [mean rating 55.5 (20.6)]. Mean arousal ratings for aversive images were 50.8 (29.6), appetitive images 58.7 (25.6), and for neutral images 34.5 (24.7). Three images, however, were rated opposite to initial valence categorization and trials including these images were therefore removed from all further analysis. To allow further analysis of valence and arousal rating, mean ratings for both appetitive and aversive images were converted to a 0–50 scale, ranging from neutral (0) to very positive/very negative (50) for appetitive and aversive images, respectively. To investigate the effect of valence ratings on RIT we performed a Bayesian correlations pair analysis between valence and RIT. The analysis revealed anecdotal evidence in favor of the absence of a correlation between the variables (*r* = −0.226, BF_01_ = 2.240) and valence ratings were therefore not considered further in subsequent analysis.

### Questionnaires

Average BIS score for the participant was 16.1 (2.7), and for the BAS subscales the average score was 9.6 (2.5) for Drive, 7.6 (1.8) for Fun seeking and 9.0 (2.5) for Reward Responsiveness. For TCI-R Persistence average score was 114.71 (14.1) and for TCI-R Harm Avoidance average score was 84.7 (13.7) (see Table 3).

**TABLE 3:** Questionnaire scores.

	BIS	BAS drive	BAS fun seeking	BAS reward responsiveness	TCI-R persistence	TCI-R harm avoidance
Mean	16.134	9.804	7.632	9.065	114.71	84.742
Std. deviation	2.655	2.495	1.76	2.502	14.117	13.709
Minimum	11	5	5	5	92	50
Maximum	22	16	12	17	144	112

## Discussion

We found that appetitive and aversive stimuli bias grip force control of distal fingers during gripping and slipping in the same way as previously described for whole-body or whole-limb movements. The results confirmed our hypothesis that there is an inherent response bias with longer response initiation times for incongruent conditions (*approach aversive* and *avoid appetitive*) compared to congruent conditions (*approach appetitive* and *avoid aversive*). Participants were consistently faster to approach appetitive stimuli than aversive stimuli and they were faster to avoid aversive stimuli than appetitive stimuli. This was reflected by the extreme evidence for a model including the interaction between response and valence, and the same pattern also clearly emerged on an individual level. In addition, most participants were slower in both incongruent conditions (*approach aversive* and *avoid appetitive*) compared to the congruent conditions (*approach positive* and a*void aversive*) regardless of the type of grip response (gripping or slipping). A similar pattern emerged for error rates, as participants committed more errors in the incongruent conditions compared to congruent conditions. Extending previous work on approach-avoidance behavior in humans, the results show for the first time that approach-avoidance biases not only movements of the entire arm or body that can span larger physical distances, but are also present when humans control the force level exerted with their fingertips during a pincer grip. Our finding thus supports the notion that the valence-bias of approach and avoidance behavior generalizes across motor effectors.

Since responses were cued by pictures depicting graspable objects or faces, we were able to test whether the individual expression of the approach-avoidance bias during gripping and slipping differed depending on cue category. Analog coding of force control has been shown to exist across physical stimulus properties: A strong stimulus intensity across different physical dimensions (loud, bright, etc.) have been shown to be more compatible with stronger force responses and low intensities have found to be more compatible with weaker responses, while the opposite mapping elicits an incongruency effect with longer reaction times (Mattes et al., 2002). Furthermore, emotional words (Solarz, 1960) and affective vocalizations have been shown to bias distal arm movements in a similar manner that emotional faces do (Koch et al., 2020). This led us to expect that grip responses should similarly be more compatible with approach responses and slip with avoid responses for graspable objects and face stimuli, even though the latter are not closely related to grasping behavior. This hypothesis was confirmed by the fact that the interaction between Response and Valence was present in both tasks (see Supplementary Tables 2, 3). Furthermore, we found no evidence for a three-way interaction between Response, Valence and Task but rather moderate evidence for the absence of such an effect. It thus appears that approach and avoidance behavior is robustly expressed during grasping and slipping movements and generalizes across visual stimulus categories. This finding lends further support to the notion of a supramodal mechanism controlling approach-avoidance behavior. Not only does the control of emotionally valent action tendencies generalize across movements as discussed above, but it also generalizes across semantic cue categories.

It could, however, be argued that faces and objects share physical properties in that faces are somehow “graspable” and the facial emotion may trigger or suppress tendencies to approach and touch the other person. In our additional analysis of the AAT-face task we found that the faces’ gender had an effect on approach-avoidance behavior. The Response by Valence interaction still was a strong effect, but there was an additional interaction with gender such that there was almost no difference in RIT between avoid responses to happy and angry female faces (being generally faster), and neither between approach responses to happy and angry male faces (being generally slow). Approaches to female faces and avoidance of male faces on the other hand showed the expected incongruency effect. This complex interaction effect was unexpected and is difficult to interpret with the given data. A future study with both male and female participants including assessments of their sexual orientation and individual scorings of the attractiveness of the face stimuli would be required to replicate the effect and understand it in more detail.

Grip-slip responses also differed according to the type of cue and response. Participants were generally faster to initiate a response to faces compared to objects. This is most likely related to difficulty levels: the facial emotional expressions depicted in our study were un-ambiguous, whereas our set of graspable objects varied in terms of how difficult they were to recognize. Participants were also faster to initiate an avoidance response compared to an approach response. This may be explained by the design of our grip force device. Even during avoid conditions, in which participants were instructed to slip, they never lost hold of the device completely but still held the handle in a light power grip. This might have modulated the perceived “risk” of dropping a positive stimulus and the “benefit” of getting rid of a negative one and this main effect of valence on RIT might be different, if a slip response entailed slipping the device entirely.

We also explored whether personality measures related to approach and avoidance behavior would be related to the strength of the incongruency effect on response initiation times in our task: Firstly, we investigated if scores on personality traits related to avoidance responses (BIS scale and TCI-R dimension harm-avoidance) would be correlated positively with response speed during avoid responses to negative stimuli. This was not supported by the data as the inclusion BF for these two measures was inconclusive with weak evidence for the absence of an effect of both measures (see Supplementary Table 4). Then, we went on to investigate personality traits associated with approach tendencies and expected high scores on the BAS scales and the TCI-R dimension persistence to be related to faster approach responses to positive stimuli. This idea was not supported for our main measure of interest; there was again weak evidence for the absence of an effect of all BAS sub-scales as well as the TCI-R dimension persistence (see Supplementary Table 4). Previous studies do not, however, provide any intuitive explanation for our finding. It does show, however, that additional variables allowed us to explain more aspects of approach and avoidance behavior that we would not be able to with RIT only as dependent variable. However, additional studies would be needed to further elucidate the relationship between personality variables and grip related approach and avoidance behavior.

### Methodological considerations

Despite robust interaction effects, some methodological aspects of the task are worth mentioning. First, the error rates found in our study were considerably higher than similar studies using a joystick. Prior to data collection, extensive piloting of the task was performed in order to establish force levels that were tenable throughout the experiment without leading to fatigue. For future studies, it might still be possible to adjust baseline force levels in order to improve performance. Second, our task demanded complex multisensory integration of the proprioceptive and the visual feedback of the produced force. Most grip force actions in real life, however, usually only involve proprioception and it is unclear how this additional complexity might have affected performance. Finally, the expanding and shrinking circle may also have produced a visual approach (zooming in) when gripping or avoidance (zooming out) experience when slipping. In the “zooming” version of the AAT (Rinck and Becker, 2007), stimuli expanded when participants pulled the joystick toward themselves, and diminished in size when they pushed it away from themselves, thus providing an ecologically valid visual effect of approaching and avoiding. Although the stimuli in the present study remained constant in size, the expansion and shrinkage of the underlying circle may have induced a similar approach or avoid experience: A “zooming in” experience caused by gripping should thereby have enhanced the “approach” context and a “zooming out” experience caused by slipping should have enhanced the “avoidance” context. Some caution regarding the generalizability across face stimuli is therefore warranted, because the incongruency effect may have been indirectly affected by the visual experience of the circle, rather than by the emotional faces (which one would usually not grip or slip) themselves. Another limitation regarding generalizability concerns the study population. Since the present study only included male young participants, our results need to be confirmed in female participants and a more elderly population.

In conclusion, we showed that the system controlling emotional approach and avoidance tendencies generalizes both across effectors and semantic categories. Future studies could still benefitting from use a wider spectrum of stimulus categories, such as landscape to explicitly test approach and avoidance representations in non-graspable stimuli. It would also be interesting to use functional brain mapping to elucidate the neural mechanisms behind such a supramodal control mechanism at the brain network level. Previous studies using emotional faces and forearm movements have found the anterior prefrontal cortex (aPFC) to play an important role in the control of approach and avoidance behavior (Volman et al., 2011, 2016; Tyborowska et al., 2016; Kaldewaij et al., 2019; Bramson et al., 2020a,b). The aPFC may therefore also be engaged in adjusting the control of force during grip force to the approach-avoidance context.

## Data availability statement

The raw data supporting the conclusions of this article will be made available by the authors, without undue reservation.

## Ethics statement

The studies involving human participants were reviewed and approved by De Videnskabsetiske Komitéer, Region Hovedstaden (H-3-2014-109). The patients/participants provided their written informed consent to participate in this study.

## Author contributions

SN developed the stimuli for AAT-Objects and HS and DM revised them and performed research. SN, DM, and KM developed the code. KM, IT, and HS discussed and revised the analyses. SN and DM wrote the manuscript, prepared the figures, and analyzed the data. All authors designed research, reviewed, and approved the figures and the manuscript.
