# Supplementary material for: Get to grips with motivation: Slipping and gripping movements are biased by approach-avoidance context
Source: Front Psychol. 2022 Oct 18;13:989495. doi: 10.3389/fpsyg.2022.989495 (PMC9623043; doi:10.3389/fpsyg.2022.989495)
Supplement: Supplementary file 1 [file Data_Sheet_1.docx]

**Supplementary figure 1.** Mean RIT for each of the conditions *(approach appetitive, approach aversive, avoid aversive* and *avoid appetitive*) in the version of the AAT depicting faces stimuli with female and male gender.

**Supplementary figure 2.** Upper section: Correlation pairs between BAS Fun seeking scores and mean PRA for each of the conditions *(approach appetitive, approach aversive, avoid aversive* and *avoid appetitive*) in the version of the AAT depicting faces as stimuli. Lower section: Correlation pairs between BAS Fun seeking scores and mean PRA for each of the conditions *(approach appetitive, approach aversive, avoid aversive* and *avoid appetitive*) in the version of the AAT depicting objects as stimuli.

**Supplementary figure 3.** Mean AUC for each of the conditions *(approach appetitive, approach aversive, avoid aversive* and *avoid appetitive*) in the two versions of the AAT.**Supplementary tables**

**Table 1.** Follow-up exploratory analysis revealed a negative correlation between scores on TCI-R and error rates for all conditions across both tasks. The only correlation that gained support (anecdotal, BF_10_ 1-3) was the one between error rates in AAT Faces for the condition *avoid positive*.

| **Bayesian Pearson Correlation** | | | | | | | | | |
| --- | --- | --- | --- | --- | --- | --- | --- | --- | --- |
|  | |  | |  | | **r** | | **BF₁₀** | |
| TCI-R_Persistence |  | - |  | er_ob_ap_pos |  | -0.111 |  | 0.264 |  |
| TCI-R_Persistence |  | - |  | er_ob_ap_neg |  | -0.299 |  | 0.797 |  |
| TCI-R_Persistence |  | - |  | er_ob_av_pos |  | -0.293 |  | 0.760 |  |
| TCI-R_Persistence |  | - |  | er_ob_av_neg |  | -0.183 |  | 0.356 |  |
| TCI-R_Persistence |  | - |  | er_face_ap_pos |  | -0.115 |  | 0.268 |  |
| TCI-R_Persistence |  | - |  | er_face_ap_neg |  | -0.239 |  | 0.498 |  |
| TCI-R_Persistence |  | - |  | er_face_av_pos |  | -0.397 |  | 2.304 |  |
| TCI-R_Persistence |  | - |  | er_face_av_neg |  | -0.199 |  | 0.387 |  |
|  | | | | | | | | | |

| **Table 2.** Model winner and BF inclusion for each of the factors in the models for AAT-Faces explaining RIT  **AAT-Faces** | | | | | | | | | | | |
| --- | --- | --- | --- | --- | --- | --- | --- | --- | --- | --- | --- |
| **Model winner** | |  | |  | |  | | **BF _10_** | |  | |
| Response + Valence + TCI-R Harm avoidance + BIS + BAS Reward responsiveness + Response  ✻  Valence |  |  |  |  |  |  |  | 2.833e +6 |  |  |  |

| **Factor** | |  | |  | | **BF _incl_** | |
| --- | --- | --- | --- | --- | --- | --- | --- |
| Response |  |  |  |  |  | 57094.830 |  |
| Valence |  |  |  |  |  | 67.189 |  |
| TCI-R_Persistence |  |  |  |  |  | 0.105 |  |
| TCI-R Harm avoidance |  |  |  |  |  | 8.671 |  |
| BIS |  |  |  |  |  | 9.353 |  |
| BAS Drive |  |  |  |  |  | 0.092 |  |
| BAS Fun seeking |  |  |  |  |  | 0.097 |  |
| BAS Reward responsiveness |  |  |  |  |  | 7.591 |  |
| Response  ✻  Valence |  |  |  |  |  | 336.761 |  |
|  | | | | | | | |

**Table 3.** Model winner and BF inclusion for each of the factors in the models for AAT-Objects explaining RIT.

| **AAT- Objects**  **Model winner** | | | | |  | | |  | | | |  | | **BF _10_** | |  | |
| --- | --- | --- | --- | --- | --- | --- | --- | --- | --- | --- | --- | --- | --- | --- | --- | --- | --- |
| Response + Valence + Response  ✻  Valence | | | |  |  |  | |  | |  | |  |  | 9403.283 |  |  |  |
|  | | | | | | | | | | | | | | | | | |
|  | | | | | | | | | | | | | | | | | |
| **Analysis of Effects AAT-Objects** | | | | | | | | | |  |  |  |  |  |  |  |  |
| **Effects** | |  | |  | | **BF _incl_** | | | |  |  |  |  |  |  |  |  |
| Response |  |  |  |  |  | | 4873.372 | |  | |  |  |  |  |  |  |  |
| Valence |  |  |  |  |  | | 134.655 | |  | |  |  |  |  |  |  |  |
| TCI-R_Persistence |  |  |  |  |  | | 1.018 | |  | |  |  |  |  |  |  |  |
| TCI-R Harm avoidance |  |  |  |  |  | | 0.776 | |  | |  |  |  |  |  |  |  |
| BIS |  |  |  |  |  | | 0.876 | |  | |  |  |  |  |  |  |  |
| BAS Drive |  |  |  |  |  | | 0.777 | |  | |  |  |  |  |  |  |  |
| BAS Fun seeking |  |  |  |  |  | | 0.739 | |  | |  |  |  |  |  |  |  |
| BAS Reward responsiveness |  |  |  |  |  | | 0.776 | |  | |  |  |  |  |  |  |  |
| Response  ✻  Valence |  |  |  |  |  | | 519.047 | |  | |  |  |  |  |  |  |  |
|  | | | | | | | | | |  |  |  |  |  |  |  |  |

**Table 4.** BF inclusion for each of the factors in the models explaining RIT.

| **Effects** | | **BF _incl_** | |
| --- | --- | --- | --- |
| Task |  | ∞ |  |
| Response |  | 53396.699 |  |
| Valence |  | 55.582 |  |
| BAS Drive |  | 0.680 |  |
| BAS Fun seeking |  | 0.667 |  |
| BAS Reward responsiveness |  | 0.661 |  |
| TCI-R_Persistence |  | 0.966 |  |
| TCI-R Harm avoidance |  | 0.840 |  |
| BIS |  | 0.662 |  |
| Task  ✻  Response |  | 0.437 |  |
| Valence  ✻  Task |  | 0.788 |  |
| Valence  ✻  Response |  | 269.420 |  |
| Valence  ✻  Task  ✻  Response |  | 0.224 |  |

**Table 5.** Bayesian Pearson Correlations between BAS dimensions and AUC.

|  |  | Face ap pos | Face ap neg | Face av pos | Face av neg | Obj ap pos | Obj ap neg | Obj av pos | Obj av neg |
| --- | --- | --- | --- | --- | --- | --- | --- | --- | --- |
| BAS Drive | r | 0.049 | 0.029 | 0.138 | 0.161 | 0.1 | 0.149 | 0.25 | 0.232 |
|  | BF₁₀ | 0.231 | 0.226 | 0.29 | 0.319 | 0.256 | 0.304 | 0.537 | 0.475 |
| BAS Reward Responsiveness | r | -0.201 | -0.169 | -0.022 | 0.145 | -0.173 | -0.171 | 0.111 | 0.28 |
|  | BF₁₀ | 0.392 | 0.331 | 0.225 | 0.298 | 0.338 | 0.335 | 0.265 | 0.678 |
| BAS Fun Seeking | r | -0.244 | -0.296 | -0.227 | -0.254 | -0.24 | -0.209 | -0.282 | -0.195 |
|  | BF₁₀ | 0.514 | 0.779 | 0.46 | 0.555 | 0.501 | 0.41 | 0.688 | 0.379 |

Obj = Object task, Face = Faces task, Ap = approach response, av = avoid response, pos = positive (appetitive) valence stimulus, neg = negative (aversive) valence stimulus
